# (*E*)-1-(4-Chloro­benzyl­idene)-2-phenyl­hydrazine

**DOI:** 10.1107/S1600536811032958

**Published:** 2011-08-27

**Authors:** M. Nawaz Tahir, Muhammad Ilyas Tariq, Riaz H. Tariq, Muhammad Sarfraz

**Affiliations:** a Department of Physics, University of Sargodha, Sargodha, Pakistan; b Department of Chemistry, University of Sargodha, Sargodha, Pakistan; c Institute of Chemical and Pharmaceutical Sciences, The University of Faisalabad, Faisalabad, Pakistan

## Abstract

The asymmetric unit of the title compound, C_13_H_11_ClN_2_, contains two geometrically distinct mol­ecules; one mol­ecule is close to planar [dihedral angle between the aromatic rings = 2.44 (18)°] and the other is twisted about the linking hydrazide group [dihedral angle = 14.08 (19)°]. In the crystal, the N—H groups do not form hydrogen bonds and the mol­ecules are linked by weak C—H⋯π inter­actions.

## Related literature

For related structures, see: Mufakkar *et al.* (2010 ▶); Shad *et al.* (2010 ▶); Yin *et al.* (2007 ▶).
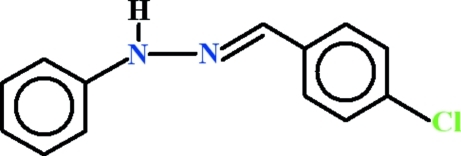


## Experimental

### 

#### Crystal data

C_13_H_11_ClN_2_
*M* *_r_* = 230.69
Orthorhombic, 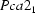

*a* = 18.6896 (9) Å
*b* = 15.0250 (7) Å
*c* = 8.4679 (4) Å
*V* = 2377.9 (2) Å^3^
*Z* = 8
Mo *K*α radiation
μ = 0.29 mm^−1^
*T* = 296 K
0.30 × 0.22 × 0.18 mm

#### Data collection

Bruker Kappa APEXII CCD diffractometer
Absorption correction: multi-scan (*SADABS*; Bruker, 2005 ▶) *T*_min_ = 0.972, *T*_max_ = 0.983
10661 measured reflections
4631 independent reflections
2234 reflections with *I* > 2σ(*I*)
*R*_int_ = 0.047

#### Refinement

*R*[*F* ^2^ > 2σ(*F* ^2^)] = 0.046
*wR*(*F* ^2^) = 0.107
*S* = 0.96
4631 reflections
295 parameters
1 restraint
H atoms treated by a mixture of independent and constrained refinement
Δρ_max_ = 0.15 e Å^−3^
Δρ_min_ = −0.12 e Å^−3^
Absolute structure: Flack (1983 ▶), 2120 Friedel pairs
Flack parameter: 0.08 (8)

### 

Data collection: *APEX2* (Bruker, 2009 ▶); cell refinement: *SAINT* (Bruker, 2009 ▶); data reduction: *SAINT*; program(s) used to solve structure: *SHELXS97* (Sheldrick, 2008 ▶); program(s) used to refine structure: *SHELXL97* (Sheldrick, 2008 ▶); molecular graphics: *ORTEP-3 for Windows* (Farrugia, 1997 ▶) and *PLATON* (Spek, 2009 ▶); software used to prepare material for publication: *WinGX* (Farrugia, 1999 ▶) and *PLATON*.

## Supplementary Material

Crystal structure: contains datablock(s) global, I. DOI: 10.1107/S1600536811032958/hb6364sup1.cif

Structure factors: contains datablock(s) I. DOI: 10.1107/S1600536811032958/hb6364Isup2.hkl

Supplementary material file. DOI: 10.1107/S1600536811032958/hb6364Isup3.cml

Additional supplementary materials:  crystallographic information; 3D view; checkCIF report

## Figures and Tables

**Table 1:** Hydrogen-bond geometry (Å, °) *Cg*1 and *Cg*2 are the centroids of the C14–C19 and C1–C6 rings, respectively.

*D*—H⋯*A*	*D*—H	H⋯*A*	*D*⋯*A*	*D*—H⋯*A*
C10—H10⋯*Cg*1^i^	0.93	2.91	3.668 (4)	139
C20—H20⋯*Cg*2^ii^	0.93	2.73	3.660 (4)	174

Symmetry codes: (i) 
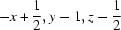
; (ii) 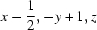.

**Fig. 1.**
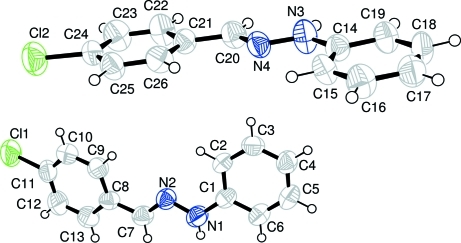
View of the title compound with displacement ellipsoids drawn at the 50% probability level.

## supplementary crystallographic information

### Comment

We have reported crystal structures of Schiff bases containing phenylhydrazine (E)-1-(2-nitrobenzylidene)-2-phenylhydrazine (Shad et al., 2010) and (E)-1-(4-methoxybenzylidene)-2-phenylhydrazine 2-nitrobenzaldehyde (Mufakkar et al., 2010). The crystal structure of (E)-N- (2,4-dichlorobenzylidene)-N^'^-phenylhydrazine (Yin, et al., 2007) has been published which is related to the title compound (I), (Fig. 1).

The title compound consists of two molecules in the crystallographic asymmetric unit which differ from each other geometrically. In one molecule, the benzene ring A (C1—C6) of phenylhydrazine and group B (C8—C13/CL1) of 4-chlorobenzaldehyde are planar with r.m.s. deviation of 0.006 and 0.018 Å, respectively. The Schiff base group C (C7/N1/N2) is of course planar. The dihedral angle between A/B, A/C and B/C is 14.62 (16)°, 8.18 (51)° and 9.86 (48)°, respectively. In second molecule, the benzene ring D (C14—C19) and group E (C21—C26) of 4-chlorobenzaldehyde are also almost planar with r.m.s. deviations of 0.004 and 0.016 Å, respectively. The dihedral angle between D/E is 2.84 (15)°. The central group F (C20/N3/N4) of this molecule makes dihedral angle of 3.10 (41)° with group D, whereas it is oriented at 0.99 (43)° with group E. This molecule is therefore, essentially planar with r.m.s. deviation of 0.026 Å. The molecules are consolidated due to van Der Wal and C—H···π interactions (Table 1).

### Experimental

Equimolar quantities of phenylhydrazine and 4-chlorobenzaldehyde were refluxed in methanol for 30 min resulting in light yellow solution. The solution was kept at room temperature which affoarded yellow prisms after 72 h.

### Refinement

The coordinates of H-atoms of N—H groups were refined. The C-bound H-atoms were positioned geometrically (C–H = 0.93 Å) and refined as riding with *U*_iso_(H) = *xU*_eq_(C, N), where *x* = 1.2 for all H-atoms.

### Crystal data

**Table:**

C_13_H_11_ClN_2_	*F*(000) = 960
*M**_r_* = 230.69	*D*_x_ = 1.289 Mg m^−^^3^
Orthorhombic, *P**c**a*2_1_	Mo *K*α radiation, λ = 0.71073 Å
Hall symbol: P 2c -2ac	Cell parameters from 2234 reflections
*a* = 18.6896 (9) Å	θ = 2.6–26.0°
*b* = 15.0250 (7) Å	µ = 0.29 mm^−^^1^
*c* = 8.4679 (4) Å	*T* = 296 K
*V* = 2377.9 (2) Å^3^	Prism, yellow
*Z* = 8	0.30 × 0.22 × 0.18 mm

### Data collection

**Table:**

Bruker Kappa APEXII CCD diffractometer	4631 independent reflections
Radiation source: fine-focus sealed tube	2234 reflections with *I* > 2σ(*I*)
graphite	*R*_int_ = 0.047
Detector resolution: 7.80 pixels mm^-1^	θ_max_ = 26.0°, θ_min_ = 2.6°
ω scans	*h* = −23→17
Absorption correction: multi-scan (*SADABS*; Bruker, 2005)	*k* = −18→8
*T*_min_ = 0.972, *T*_max_ = 0.983	*l* = −10→10
10661 measured reflections	

### Refinement

**Table:**

Refinement on *F*^2^	Secondary atom site location: difference Fourier map
Least-squares matrix: full	Hydrogen site location: inferred from neighbouring sites
*R*[*F*^2^ > 2σ(*F*^2^)] = 0.046	H atoms treated by a mixture of independent and constrained refinement
*wR*(*F*^2^) = 0.107	*w* = 1/[σ^2^(*F*_o_^2^) + (0.034*P*)^2^] where *P* = (*F*_o_^2^ + 2*F*_c_^2^)/3
*S* = 0.96	(Δ/σ)_max_ < 0.001
4631 reflections	Δρ_max_ = 0.15 e Å^−^^3^
295 parameters	Δρ_min_ = −0.12 e Å^−^^3^
1 restraint	Absolute structure: Flack (1983), 2120 Friedel pairs
Primary atom site location: structure-invariant direct methods	Flack parameter: 0.08 (8)

### Special details

**Table:**

Geometry. Bond distances, angles *etc*. have been calculated using the rounded fractional coordinates. All su's are estimated from the variances of the (full) variance-covariance matrix. The cell e.s.d.'s are taken into account in the estimation of distances, angles and torsion angles
Refinement. Refinement of *F*^2^ against ALL reflections. The weighted *R*-factor *wR* and goodness of fit *S* are based on *F*^2^, conventional *R*-factors *R* are based on *F*, with *F* set to zero for negative *F*^2^. The threshold expression of *F*^2^ > σ(*F*^2^) is used only for calculating *R*-factors(gt) *etc*. and is not relevant to the choice of reflections for refinement. *R*-factors based on *F*^2^ are statistically about twice as large as those based on *F*, and *R*- factors based on ALL data will be even larger.

### Fractional atomic coordinates and isotropic or equivalent isotropic displacement parameters (Å^2^)

**Table:**

	*x*	*y*	*z*	*U*_iso_*/*U*_eq_	
Cl1	0.65074 (8)	−0.28852 (7)	0.04443 (17)	0.1121 (6)	
N1	0.7409 (2)	0.2275 (3)	0.1957 (4)	0.0747 (16)	
N2	0.71259 (15)	0.1473 (2)	0.1565 (3)	0.0620 (12)	
C1	0.7116 (2)	0.3043 (3)	0.1339 (4)	0.0570 (17)	
C2	0.6470 (2)	0.3045 (2)	0.0531 (5)	0.0600 (16)	
C3	0.6199 (2)	0.3839 (3)	−0.0015 (4)	0.0680 (16)	
C4	0.6544 (2)	0.4633 (3)	0.0205 (5)	0.0700 (17)	
C5	0.7189 (2)	0.4618 (3)	0.0988 (4)	0.0733 (17)	
C6	0.7466 (2)	0.3843 (3)	0.1566 (5)	0.0667 (16)	
C7	0.7460 (2)	0.0782 (3)	0.2017 (4)	0.0660 (17)	
C8	0.7209 (2)	−0.0111 (3)	0.1679 (5)	0.0610 (16)	
C9	0.6539 (2)	−0.0276 (3)	0.1025 (4)	0.0690 (17)	
C10	0.6317 (2)	−0.1110 (3)	0.0677 (4)	0.0733 (17)	
C11	0.6769 (3)	−0.1823 (3)	0.0953 (5)	0.0763 (19)	
C12	0.7428 (3)	−0.1686 (3)	0.1634 (6)	0.0880 (19)	
C13	0.7642 (2)	−0.0834 (3)	0.1987 (5)	0.0830 (17)	
Cl2	0.05113 (6)	0.19759 (6)	0.67491 (15)	0.0891 (5)	
N3	0.09142 (18)	0.7174 (2)	0.4688 (4)	0.0713 (14)	
N4	0.07889 (14)	0.6368 (2)	0.5353 (3)	0.0569 (11)	
C14	0.07671 (18)	0.7951 (2)	0.5497 (4)	0.0550 (14)	
C15	0.0503 (2)	0.7956 (3)	0.7018 (4)	0.0627 (17)	
C16	0.0368 (2)	0.8752 (3)	0.7763 (5)	0.0723 (17)	
C17	0.0484 (2)	0.9564 (3)	0.7053 (5)	0.0727 (17)	
C18	0.0740 (2)	0.9553 (3)	0.5522 (5)	0.0740 (19)	
C19	0.0884 (2)	0.8767 (3)	0.4756 (5)	0.0670 (17)	
C20	0.09573 (19)	0.5686 (3)	0.4543 (4)	0.0613 (16)	
C21	0.08497 (18)	0.4782 (2)	0.5112 (4)	0.0527 (14)	
C22	0.1035 (2)	0.4073 (3)	0.4174 (4)	0.0730 (16)	
C23	0.0947 (2)	0.3208 (3)	0.4663 (5)	0.0763 (17)	
C24	0.0658 (2)	0.3056 (3)	0.6128 (4)	0.0580 (16)	
C25	0.0479 (2)	0.3741 (3)	0.7092 (4)	0.0617 (16)	
C26	0.05611 (19)	0.4603 (2)	0.6581 (5)	0.0590 (14)	
H1	0.782 (2)	0.228 (2)	0.231 (5)	0.0899*	
H2	0.62234	0.25154	0.03615	0.0718*	
H3	0.57649	0.38355	−0.05530	0.0817*	
H4	0.63493	0.51639	−0.01617	0.0842*	
H5	0.74414	0.51457	0.11255	0.0878*	
H6	0.78957	0.38538	0.21195	0.0798*	
H7	0.78820	0.08512	0.25863	0.0790*	
H9	0.62358	0.02012	0.08204	0.0825*	
H10	0.58637	−0.12036	0.02547	0.0879*	
H12	0.77257	−0.21656	0.18531	0.1056*	
H13	0.80890	−0.07435	0.24434	0.0998*	
H3A	0.113 (2)	0.720 (2)	0.369 (4)	0.0858*	
H15	0.04156	0.74218	0.75390	0.0750*	
H16	0.01907	0.87411	0.87893	0.0867*	
H17	0.03937	1.00953	0.75794	0.0874*	
H18	0.08167	1.00894	0.49998	0.0888*	
H19	0.10607	0.87791	0.37294	0.0803*	
H20	0.11570	0.57692	0.35474	0.0736*	
H22	0.12260	0.41816	0.31781	0.0874*	
H23	0.10806	0.27364	0.40153	0.0915*	
H25	0.03018	0.36272	0.80979	0.0739*	
H26	0.04206	0.50705	0.72297	0.0710*	

### Atomic displacement parameters (Å^2^)

**Table:**

	*U*^11^	*U*^22^	*U*^33^	*U*^12^	*U*^13^	*U*^23^
Cl1	0.1692 (12)	0.0627 (8)	0.1044 (9)	−0.0202 (8)	−0.0151 (10)	0.0053 (7)
N1	0.067 (3)	0.063 (2)	0.094 (3)	−0.002 (2)	−0.019 (2)	−0.004 (2)
N2	0.063 (2)	0.056 (2)	0.067 (2)	0.0003 (18)	−0.0010 (19)	−0.003 (2)
C1	0.055 (3)	0.060 (3)	0.056 (3)	−0.003 (2)	0.006 (2)	−0.013 (2)
C2	0.062 (3)	0.056 (3)	0.062 (2)	−0.009 (2)	−0.005 (2)	−0.010 (2)
C3	0.070 (3)	0.072 (3)	0.062 (2)	0.004 (2)	−0.012 (2)	−0.003 (2)
C4	0.087 (3)	0.056 (3)	0.067 (3)	−0.001 (2)	0.002 (2)	−0.010 (2)
C5	0.079 (3)	0.064 (3)	0.077 (3)	−0.017 (2)	0.011 (2)	−0.016 (2)
C6	0.057 (2)	0.064 (3)	0.079 (3)	−0.005 (2)	−0.001 (2)	−0.012 (3)
C7	0.060 (3)	0.068 (3)	0.070 (3)	0.004 (3)	−0.008 (2)	−0.004 (2)
C8	0.056 (3)	0.065 (3)	0.062 (2)	0.004 (2)	−0.006 (2)	−0.002 (2)
C9	0.069 (3)	0.061 (3)	0.077 (3)	0.007 (2)	−0.006 (2)	0.000 (2)
C10	0.077 (3)	0.074 (3)	0.069 (3)	−0.001 (3)	−0.007 (2)	0.002 (3)
C11	0.101 (4)	0.062 (3)	0.066 (3)	−0.015 (3)	0.000 (3)	0.009 (2)
C12	0.100 (4)	0.058 (3)	0.106 (3)	0.011 (3)	−0.009 (3)	0.011 (3)
C13	0.070 (3)	0.072 (3)	0.107 (3)	0.008 (3)	−0.023 (3)	0.013 (3)
Cl2	0.1246 (9)	0.0516 (7)	0.0911 (8)	−0.0030 (6)	−0.0040 (8)	0.0068 (7)
N3	0.104 (3)	0.049 (2)	0.061 (2)	0.0020 (19)	0.0193 (19)	0.0028 (18)
N4	0.068 (2)	0.044 (2)	0.0586 (19)	0.0062 (16)	−0.0039 (17)	0.0035 (17)
C14	0.060 (2)	0.049 (3)	0.056 (2)	0.0028 (19)	0.001 (2)	0.000 (2)
C15	0.077 (3)	0.051 (3)	0.060 (3)	0.007 (2)	0.004 (2)	0.013 (2)
C16	0.084 (3)	0.063 (3)	0.070 (3)	0.009 (2)	0.012 (2)	−0.003 (2)
C17	0.088 (3)	0.060 (3)	0.070 (3)	−0.004 (2)	−0.001 (2)	0.007 (2)
C18	0.088 (3)	0.041 (3)	0.093 (4)	−0.004 (2)	−0.003 (3)	0.014 (2)
C19	0.076 (3)	0.057 (3)	0.068 (3)	−0.002 (2)	0.011 (2)	0.014 (2)
C20	0.074 (3)	0.057 (3)	0.053 (2)	0.004 (2)	0.004 (2)	0.000 (2)
C21	0.062 (3)	0.043 (2)	0.053 (2)	0.0034 (18)	0.0001 (19)	−0.0053 (19)
C22	0.099 (3)	0.060 (3)	0.060 (2)	0.006 (2)	0.018 (2)	−0.001 (2)
C23	0.102 (3)	0.059 (3)	0.068 (3)	0.014 (2)	0.010 (2)	−0.012 (2)
C24	0.069 (3)	0.042 (2)	0.063 (3)	−0.002 (2)	−0.010 (2)	0.0058 (19)
C25	0.081 (3)	0.053 (3)	0.051 (2)	0.001 (2)	0.003 (2)	0.003 (2)
C26	0.069 (2)	0.056 (3)	0.052 (2)	0.012 (2)	0.003 (2)	−0.011 (2)

### Geometric parameters (Å, °)

**Table:**

Cl1—C11	1.724 (5)	C7—H7	0.9300
Cl2—C24	1.728 (4)	C9—H9	0.9300
N1—N2	1.357 (5)	C10—H10	0.9300
N1—C1	1.380 (6)	C12—H12	0.9300
N2—C7	1.271 (5)	C13—H13	0.9300
N1—H1	0.82 (4)	C14—C19	1.395 (5)
N3—C14	1.381 (4)	C14—C15	1.379 (5)
N3—N4	1.356 (4)	C15—C16	1.376 (6)
N4—C20	1.273 (5)	C16—C17	1.377 (6)
N3—H3A	0.94 (3)	C17—C18	1.382 (6)
C1—C6	1.382 (6)	C18—C19	1.374 (6)
C1—C2	1.388 (5)	C20—C21	1.455 (5)
C2—C3	1.376 (5)	C21—C26	1.382 (5)
C3—C4	1.369 (6)	C21—C22	1.373 (5)
C4—C5	1.376 (5)	C22—C23	1.374 (6)
C5—C6	1.365 (6)	C23—C24	1.372 (5)
C7—C8	1.450 (6)	C24—C25	1.356 (6)
C8—C13	1.380 (6)	C25—C26	1.374 (5)
C8—C9	1.392 (5)	C15—H15	0.9300
C9—C10	1.353 (6)	C16—H16	0.9300
C10—C11	1.384 (6)	C17—H17	0.9300
C11—C12	1.376 (8)	C18—H18	0.9300
C12—C13	1.374 (6)	C19—H19	0.9300
C2—H2	0.9300	C20—H20	0.9300
C3—H3	0.9300	C22—H22	0.9300
C4—H4	0.9300	C23—H23	0.9300
C5—H5	0.9300	C25—H25	0.9300
C6—H6	0.9300	C26—H26	0.9300
			
N2—N1—C1	119.7 (3)	C11—C12—H12	120.00
N1—N2—C7	117.4 (3)	C13—C12—H12	120.00
N2—N1—H1	117 (2)	C12—C13—H13	119.00
C1—N1—H1	120 (2)	C8—C13—H13	119.00
N4—N3—C14	121.0 (3)	N3—C14—C15	122.6 (3)
N3—N4—C20	116.9 (3)	N3—C14—C19	119.3 (3)
C14—N3—H3A	119.8 (19)	C15—C14—C19	118.1 (3)
N4—N3—H3A	119.1 (19)	C14—C15—C16	119.9 (4)
N1—C1—C6	119.1 (4)	C15—C16—C17	122.8 (4)
N1—C1—C2	122.3 (4)	C16—C17—C18	117.0 (4)
C2—C1—C6	118.6 (4)	C17—C18—C19	121.4 (4)
C1—C2—C3	119.2 (3)	C14—C19—C18	120.8 (4)
C2—C3—C4	122.4 (4)	N4—C20—C21	122.6 (3)
C3—C4—C5	117.7 (4)	C22—C21—C26	117.9 (3)
C4—C5—C6	121.3 (4)	C20—C21—C22	119.9 (3)
C1—C6—C5	120.8 (4)	C20—C21—C26	122.2 (3)
N2—C7—C8	122.5 (3)	C21—C22—C23	122.0 (3)
C7—C8—C9	122.3 (4)	C22—C23—C24	118.5 (4)
C7—C8—C13	120.1 (4)	Cl2—C24—C25	119.4 (3)
C9—C8—C13	117.6 (4)	Cl2—C24—C23	119.6 (3)
C8—C9—C10	121.9 (4)	C23—C24—C25	121.0 (4)
C9—C10—C11	119.5 (4)	C24—C25—C26	119.9 (3)
Cl1—C11—C12	119.8 (4)	C21—C26—C25	120.7 (3)
C10—C11—C12	120.1 (4)	C14—C15—H15	120.00
Cl1—C11—C10	120.1 (4)	C16—C15—H15	120.00
C11—C12—C13	119.4 (4)	C15—C16—H16	119.00
C8—C13—C12	121.5 (4)	C17—C16—H16	119.00
C3—C2—H2	120.00	C16—C17—H17	122.00
C1—C2—H2	120.00	C18—C17—H17	122.00
C2—C3—H3	119.00	C17—C18—H18	119.00
C4—C3—H3	119.00	C19—C18—H18	119.00
C3—C4—H4	121.00	C14—C19—H19	120.00
C5—C4—H4	121.00	C18—C19—H19	120.00
C4—C5—H5	119.00	N4—C20—H20	119.00
C6—C5—H5	119.00	C21—C20—H20	119.00
C5—C6—H6	120.00	C21—C22—H22	119.00
C1—C6—H6	120.00	C23—C22—H22	119.00
C8—C7—H7	119.00	C22—C23—H23	121.00
N2—C7—H7	119.00	C24—C23—H23	121.00
C8—C9—H9	119.00	C24—C25—H25	120.00
C10—C9—H9	119.00	C26—C25—H25	120.00
C11—C10—H10	120.00	C21—C26—H26	120.00
C9—C10—H10	120.00	C25—C26—H26	120.00
			
C1—N1—N2—C7	172.3 (3)	C9—C10—C11—C12	2.5 (6)
N2—N1—C1—C2	10.7 (5)	C10—C11—C12—C13	−2.2 (7)
N2—N1—C1—C6	−171.1 (3)	Cl1—C11—C12—C13	177.8 (4)
N1—N2—C7—C8	179.6 (3)	C11—C12—C13—C8	0.3 (7)
N4—N3—C14—C19	−178.2 (3)	N3—C14—C15—C16	179.9 (3)
C14—N3—N4—C20	−178.2 (3)	C19—C14—C15—C16	−0.5 (5)
N4—N3—C14—C15	1.4 (5)	N3—C14—C19—C18	179.7 (3)
N3—N4—C20—C21	−179.8 (3)	C15—C14—C19—C18	0.0 (5)
N1—C1—C2—C3	178.2 (4)	C14—C15—C16—C17	0.1 (6)
N1—C1—C6—C5	−179.3 (4)	C15—C16—C17—C18	0.7 (6)
C2—C1—C6—C5	−1.0 (6)	C16—C17—C18—C19	−1.1 (6)
C6—C1—C2—C3	−0.1 (6)	C17—C18—C19—C14	0.8 (6)
C1—C2—C3—C4	0.2 (6)	N4—C20—C21—C22	179.3 (3)
C2—C3—C4—C5	0.8 (6)	N4—C20—C21—C26	−0.5 (5)
C3—C4—C5—C6	−1.9 (6)	C20—C21—C22—C23	179.7 (3)
C4—C5—C6—C1	2.0 (6)	C26—C21—C22—C23	−0.6 (5)
N2—C7—C8—C13	170.3 (4)	C20—C21—C26—C25	−178.8 (3)
N2—C7—C8—C9	−8.9 (6)	C22—C21—C26—C25	1.4 (5)
C13—C8—C9—C10	−0.9 (6)	C21—C22—C23—C24	0.7 (6)
C7—C8—C13—C12	−178.0 (4)	C22—C23—C24—Cl2	177.7 (3)
C7—C8—C9—C10	178.3 (4)	C22—C23—C24—C25	−1.6 (6)
C9—C8—C13—C12	1.3 (6)	Cl2—C24—C25—C26	−176.9 (3)
C8—C9—C10—C11	−1.0 (6)	C23—C24—C25—C26	2.5 (6)
C9—C10—C11—Cl1	−177.5 (3)	C24—C25—C26—C21	−2.4 (6)

### Hydrogen-bond geometry (Å, °)

**Table:**

Cg1 and Cg2 are the centroids of the C14–C19 and C1–C6 rings, respectively.

**Table:**

*D*—H···*A*	*D*—H	H···*A*	*D*···*A*	*D*—H···*A*
C10—H10···Cg1^i^	0.93	2.91	3.668 (4)	139
C20—H20···Cg2^ii^	0.93	2.73	3.660 (4)	174

Symmetry codes: (i) −*x*+1/2, *y*−1, *z*−1/2; (ii) *x*−1/2, −*y*+1, *z*.
